# Immunohistochemical Characterization of Tumor-Associated Macrophages in Canine Lymphomas

**DOI:** 10.3390/ani11082301

**Published:** 2021-08-04

**Authors:** Sergio Vázquez, Raquel Vallejo, José Espinosa, Noive Arteche, José A. Vega, Valentín Pérez

**Affiliations:** 1 Departamento de Sanidad Animal, Universidad de León, 24007 León, Spain; rvalg@unileon.es (R.V.); jespic@unileon.es (J.E.); nartv@unileon.es (N.A.); vperp@unileon.es (V.P.); 2 Grupo SINPOS, Departamento de Morfología y Biología Celular, Universidad de Oviedo, 33006 Oviedo, Spain; javega@uniovi.es; 3 Facultad de Ciencias de la Salud, Universidad Autónoma de Chile, Providencia 7500912, Santiago de Chile, Chile

**Keywords:** canine, histology, immunohistochemistry, lymphoma, macrophages

## Abstract

**Simple Summary:**

In human lymphomas, it has been shown that the macrophages present in the tumor have an influence on their behavior; however, studies on this topic are scarce in veterinary medicine. The aim of this work was to determine the relationship between the amount and type of macrophage infiltrates with histological grade and immunophenotype in cases of canine lymphoma. Samples from the lymph nodes of 25 dogs with lymphoma were analyzed. Immunohistochemistry was used to determine the tumor immunophenotype (CD3 and CD20 antibodies) and the macrophage characterization (Iba1, CD163, iNOS and MAC387 antibodies). Results showed that the highest number of macrophages were found in high-grade and B-cell lymphomas; the latter also presented the greatest amount of M1 and M2 macrophages. High-grade lymphomas showed a greater number of M2 and recently recruited macrophages that were most abundant in T than in B-cell lymphomas. In conclusion, the number and type of macrophages present in canine lymphoma are related to the immunophenotype and the grade. In those with a high grade, macrophages are actively recruited and show a predominant M2 phenotype, which has been associated with a protumoral activity.

**Abstract:**

Macrophages have been confirmed to play a significant role in the behavior of human lymphomas, albeit no consistent data are so far available in canine lymphomas. The present study characterizes the macrophages present in cases of canine nodal lymphoma and their relationship with the histological grade and the immunophenotype. Samples from the lymph nodes of 25 dogs diagnosed with lymphoma were selected. Immunohistochemistry was used to determine the tumor immunophenotype (CD3 and CD20 antibodies) and macrophage characterization (Iba1, MAC387, CD204, CD163 and iNOS antibodies). Macrophage counting was performed in 10 randomly selected, high-power fields per sample. Generalized linear models with Poisson distribution were used for statistical analysis. A significantly greater number of macrophages (Iba1+) were detected in high-grade and B-cell lymphomas. The highest amount of both M1 (iNOS+) and M2 (CD204+ and CD163+) subtypes were observed in B-cell lymphomas. High-grade lymphomas showed a greater number of CD204+ and CD163+ cells and recently recruited MAC387+ macrophages. The latter were most abundant in T than in B-cell lymphomas. In conclusion, a significant population of macrophages is present in canine lymphomas, which constitute a heterogeneous population that shows variations in the amount and immunohistochemical profile according to the histological grade and immunophenotype.

## 1. Introduction

Non-tumor cells present in the tissues affected by a neoplasm play an essential role in the development and spread of a tumor so that, among other functions, it can allow the host immune system evasion, directly or indirectly, by the release of modulating major bioactive molecules [1]. The set of cells that interact with the neoplasm determines the tumor microenvironment and are of capital importance in the behavior, pathogenesis and prognosis of the tumor, as well as the response to treatment [2].

Tumor-associated macrophages (TAM) are one of the main cells responsible for the maintenance of the tumor microenvironment. They represent the main escape route of the neoplasia by the active suppression of the immune system and the modulation of other tumor microenvironment cell types [3,4]. TAM are classically classified into two main types. On the one hand, M1 macrophages present an antitumoral function, promoting an inflammatory response. On the other hand, M2 macrophages have a protumoral role, contributing to the immune evasion, angiogenesis and invasion [5]. Commonly in the neoplastic tissue, both types of macrophages are identified by immunohistochemical methods. Expression of iNOS is considered to occur in M1 macrophages, while CD204 and CD163 have been used as markers for M2 macrophages, although some studies have suggested that the latter it is not highly specific for this macrophage subtype [6,7,8]. Besides this, the expression of calprotectin, a protein recognized by the MAC387 antibody, has been used as a marker for macrophages that have recently been recruited from blood circulation and have just reached the tissue [9,10]. In the majority of the human tumors, including some types of lymphoma, M2 predominate over M1 macrophages and this difference is related to their histological grade (HG) [11,12,13,14].

Lymphoma is one of the most frequent neoplasms in both humans and dogs, representing 80–85% of hematopoietic neoplasms and approximately 8–9% of all tumors in the canine species [15]. It is originated by the neoplastic proliferation of cells of lymphoid origin, which occurs more frequently in lymphoid tissues such as lymph nodes, spleen or bone marrow, but other locations are affected such as the liver or the nervous system [16]. In this particular type of tumors, macrophages are commonly found scattered among the neoplastic lymphocytes. In human lymphomas, especially in diffuse large B-cell lymphomas (DLBCL), there is a negative correlation between the number of M2 macrophages present in the tumor and the prognosis [13,17]. This is due to the protumoral action of M2—CD204+ macrophages (with an anti-inflammatory function) that are associated with an increased tumor invasion and ability to spread [13,14]. By contrast, M1—iNOS+ macrophages play an antitumoral role (proinflammatory function) [5,18,19,20]. Similar results have been found in other tumors different from lymphoma in the canine species [21,22,23]. Regarding the immunophenotype (IPT) of lymphomas, data about what cytokines are produced in type B or type T to attract macrophages to the tumor are only partially known, and the knowledge of the predominance of the different macrophages in the lymphoma types still remains unknown [2,24,25,26].

Regarding veterinary medicine, only few studies have evaluated the presence of macrophages using immunohistochemical methods in tumors in the canine species [3,10,14,23,27,28]. Particularly and to the authors’ knowledge, no precise studies have assessed the distribution of these cells in canine lymphomas despite the importance of this type of tumor in terms of prevalence, and the studies that exist have assessed the level of monocytes in relation to lymphoma but not tissue macrophages [29]. Therefore, the main objective of this work is to characterize, by immunohistochemical methods, the macrophages present in histologically diagnosed cases of canine nodal lymphomas using several markers for the different subpopulations of these cells, together with their distribution and relationship with the HG and IPT.

## 2. Materials and Methods

### 2.1. Case Selection

Histologically diagnosed cases of non-treated canine lymphoma (*n* = 25) were selected from necropsies and biopsies analyzed at the Pathology Department of the Veterinary Teaching Hospital of the University of León. No experimental procedures have been conducted on any animal included in the study. All were natural cases of the disease managed by veterinary practitioners under regular clinical conditions and all the cases had the consent of the owners according to the protocols of the institution. The selection criteria were based on the quality of the tissues and the organs affected. In order to avoid location variations, only lymph node samples were chosen.

### 2.2. Histopathology

The tissue samples were fixed in 10% neutral buffered formalin, routinely embedded in paraffin, and cut sections (2.5 μm thick) were obtained and stained with hematoxylin and eosin (H&E). Only one lymph node sample per animal was selected, according to their quality and histological features. All the studies were always conducted in the same sample and reviewed by a board-certified veterinary pathologist (VP).

The mitotic index (MI) was estimated as an indicator of the HG [30] by counting the number of total mitoses in 10 high-powered fields (HPF, objective 40× and ocular 10×), and the grade of differentiation. The sections with an MI less than or equal to 10 mitoses were classified as low-grade lymphomas, and those with an MI greater than 10 mitoses were considered as high-grade lymphomas [30].

The histological subtype of each case of lymphoma was determined by the evaluation of their immunophenotype, as determined by immunohistochemical staining (see below) together with their morphological features, according to the guidelines provided in previous classifications [31].

### 2.3. Immunohistochemistry

Formalin-fixed, paraffin-embedded tissues were cut into 2.5 μm thick sections with a rotation microtome (Thermo Shandon Finesse^®^, Thermo Scientific, Waltham, MA, USA) and mounted on electrocharged adhesive gelatin-coated microscope slides (Thermo Scientific, Waltham, MA, USA). Then, they were routinely deparaffinized and rehydrated.

Epitope retrieval, when necessary, was carried out using the PTLink^®^ system (Agilent Corporation, Santa Clara, CA, USA). The endogenous peroxidase was inactivated using a solution of 3% hydrogen peroxide in methanol for 30 min in a humidified chamber. Primary antibodies were employed following the indications exposed in Table 1 and incubated overnight at 4 °C in a humid chamber. All of them were previously reported for their use in the canine species as markers for mentioned target macrophage and lymphocyte subpopulation [14,32,33,34,35].

Immunodetection was performed using the EnVision^®^ + Rabbit polymer or the EnVision^®^ + Mouse polymer (Agilent Corporation, Santa Clara, CA, USA) when appropriate, incubating the sections for 40 min at room temperature in a humid chamber. Immunoreactivity was visualized using a diaminobenzidine (Agilent Corporation, Santa Clara, CA, USA) as a chromogen, and thereafter the sections were counterstained with hematoxylin, dehydrated and coverslipped. Canine lymph nodes from healthy dogs were used as positive controls. The specificity of the immunoreaction was assessed by processing representative sections in the same way as described above but omitting the primary antibodies, EnVision^®^ polymers or diaminobenzidine in the incubations, and the substitution of the primary antibody with the adequate isotype-matched murine or rabbit immunoglobulin. Under these conditions no specific immunoreactivity was detected.

### 2.4. Evaluation of Immunohistochemical Expression

The determination of IPT was performed by reviewing slides stained with H&E alongside slides immunostained for CD3 and CD20 markers, and according to the proportion and distribution of cells, positively immunolabeled for each antibody [31].

Macrophage amount determination was carried out by counting manually the number of positively immunolabeled cells for each antibody in 10 HPF per slide and marker (Iba1, MAC387, CD204, CD163 and iNOS) using an image analysis program (Image J program^®^, U.S. National Institutes of Health, Bethesda, MD, USA).

### 2.5. Statistical Analysis

The normality of the data was determined using the Kolmogorov–Smirnov test, showing that all variables were non-normally distributed. The results of the counts of the different macrophage subtypes according to their HG and IPT were expressed as median and range, calculated using conventional descriptive statistical procedures.

To analyze the relative effect of the HG (high or low) and the IPT (T or B) on the number of cells expressing the different macrophage subtype markers (Iba1+, MAC387+, CD204+, CD163+ and iNOS+), generalized linear models (GLMs) with Poisson distribution, which is recommended for cell counts [36], were used. The *dredge, get.models and model.sel* functions included in the R package “MuMIn”, were employed to construct a set of candidate models with all the possible combinations of predictive variables and, according to them, we identified the best models using an automatic selection procedure based on the Akaike Information Criterion corrected for small sample sizes (AIC_c_) [37] provided by the *dredge* function. The most strong and parsimonious model was selected using the combination of lower Akaike’s Information Criterion (AIC_c_) and the Akaike weight (W_i_). Subsequently, we estimated the Akaike weight (W_i_) and the percentage of explained variability (R^2^) for each selected model [37]. The fit quality of the selected models was evaluated through the analysis of residual deviance, linearity, multicollinearity and overdispersion using diagnostic graphics (R packages “mctest” and “car”) [38,39]. To observe the differences between groups within the relevant variables included in the final fitted model, we used the Tukey’s Honestly Significant Difference adjustment for the whole pairwise comparisons using the *glht.function* with the “multcomp” package in R [40]. *p* values of less than 0.05 were considered statistically significant. Finally, the Spearman’s rank correlation coefficient test was applied in order to establish the coefficient among the different markers analyzed.

All the statistical analyses were performed with the R Software version 3.3.2 [41].

## 3. Results

### 3.1. Determination of HG and Subtype and IPT

From the 25 cases of canine nodal lymphoma included in the study, a total of 7 cases were classified as high-grade lymphomas, and the remaining 18 as low-grade lymphomas.

According to the immunohistochemical labeling of neoplastic cells with antibodies against CD3 and CD20 receptors, 14 and 11 cases were categorized as T- or B-cell lymphomas, respectively. All the 11 cases of B-cell lymphoma were classified as DLBCL. However, among the 14 cases of T-cell lymphoma, 11 were determined to be peripheral T-cell lymphomas (PTCL) and the remaining 3 cases, as T-zone lymphomas (TZL).

Among the 14 cases of T-cell lymphoma, 3 showed a high grade (all PTCL) and the remaining 11 a low grade, whereas 4 were a high grade and 7 a low grade among those cases classified as B-cell lymphomas (DLBCL).

### 3.2. Immunohistochemical Characterization of Macrophages

According to our model selection procedure, the most strong and parsimonious model, based on the fit quality indicators AIC (model with substantial support: ΔAICc <2 unit given the data set) and Wi (Akaike weight of the model) (Table S1), included an additive combination and its interaction of all explanatory variables considered for these markers (HG+IPT+HG*IPT). Results of the post-hoc analysis and *p* values are showed in Table 2. Estimation of the adjusted R2 of the fitted model was high and explained 65.7% of the variability in the number of Iba1 positively immunolabeled cells and between 99.7% and 97.3% for the rest of the markers (Table S1), with an average *p* value less than 0.01.

**Figure 1:**
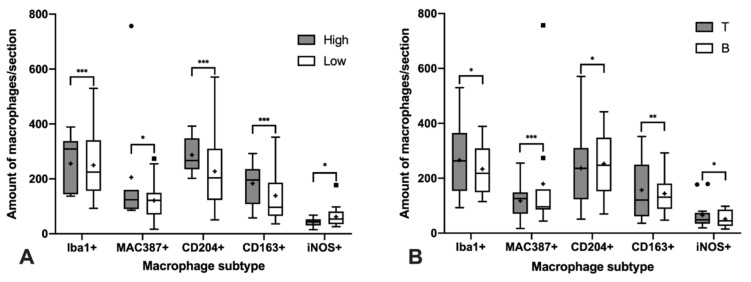
Significant differences in the median of the amount of macrophage subtypes per sample according to the histological grade (**A**) and immunophenotype (**B**). Black squares and circles represent outlayer data. *, *p* < 0.05; **, *p* < 0.01; ***, *p* < 0.001.

**Figure 2:**
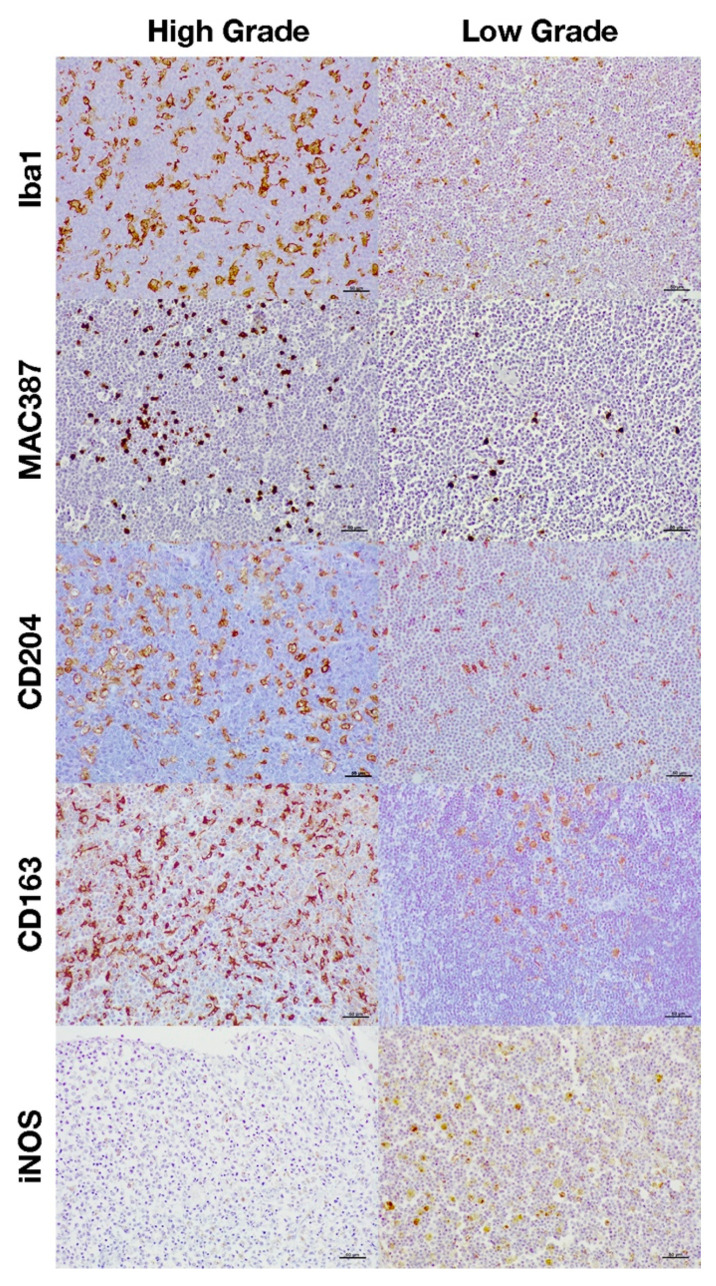
Immunohistochemical expression of the different macrophage markers according to the histological grade.

**Figure 3:**
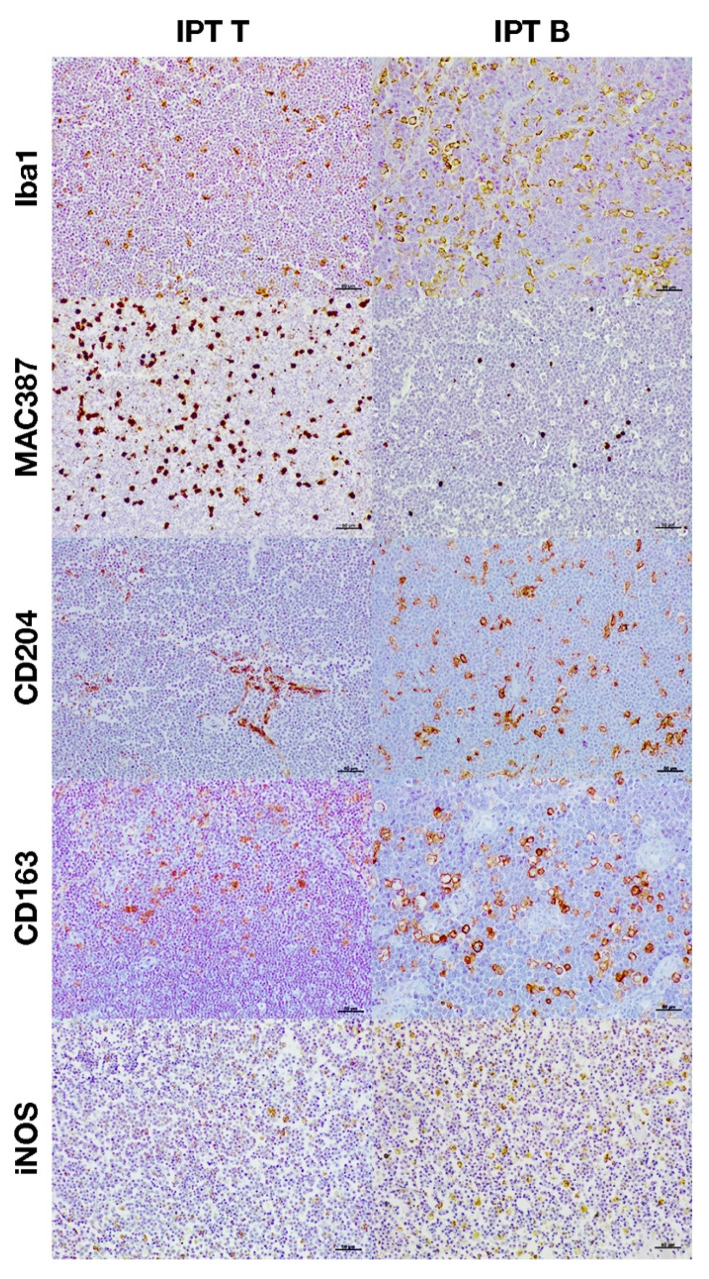
Immunohistochemical expression of the different macrophage markers according to the immunophenotype.

**Figure 4:**
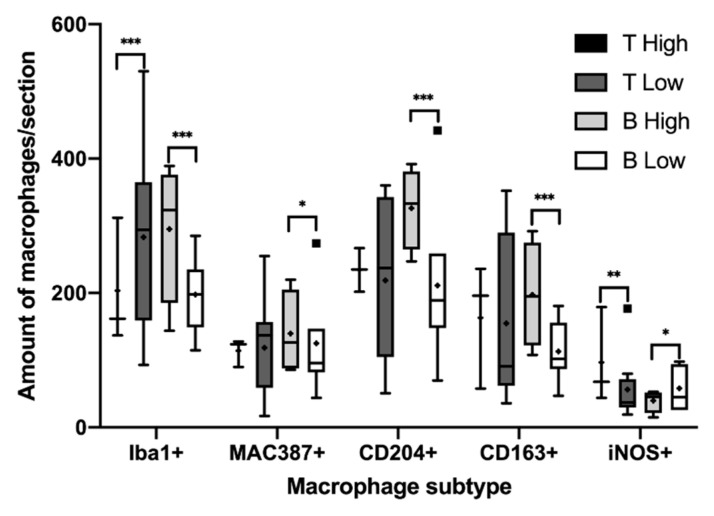
Significant differences in the median of the amount of macrophage subtypes per sample according to both the histological grade and immunophenotype. Black squares and circles represent outlayer data. *, *p* < 0.05; **, *p* < 0.01; ***, *p* < 0.001.

**Figure 5:**
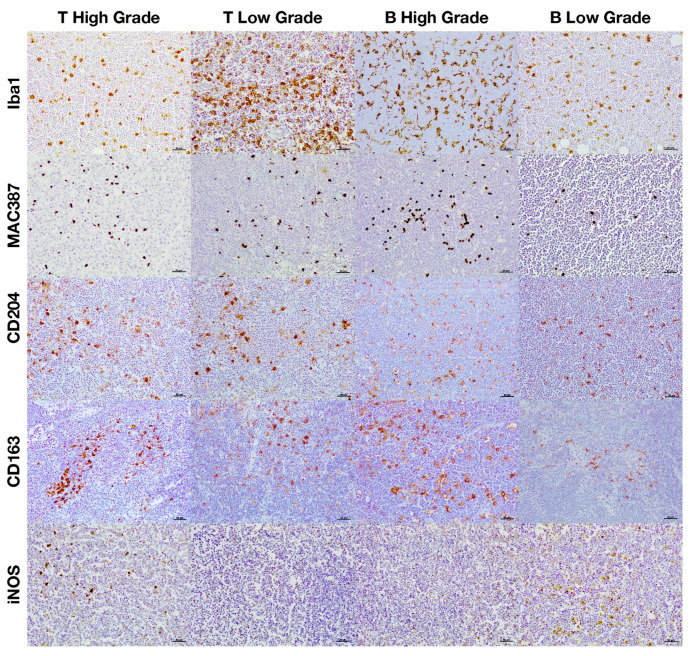
Immunohistochemical expression of the different macrophage markers according to both the histological grade and immunophenotype.

The total number of macrophages was assessed by counting those cells positively immunolabeled with Iba1 and MAC387 antibodies and the results are shown in Figure 1A significantly higher number of cells immunolabeled for both markers were detected in high-grade lymphomas (Figure 1A and Figure 2). Regarding the IPT, while T-cell lymphomas showed a significant reduction in the number of Iba1 positively immunolabeled cells with respect to B-cell lymphomas, the contrary was found when the number of cells immunostained with the MAC387 antibody was assessed (Figure 1B and Figure 3). The joint analysis between the different IPTs and HGs showed significant variation in the expression of each marker, as can be seen in Figure 4 and Figure 5.

M2 macrophages were identified by the immunohistochemical labeling with antibodies against CD204 and CD163 receptors (Figure 1). As for the Iba1 and MAC387 markers, the highest number of CD204 and CD163 immunolabeled cells was observed in high-grade lymphomas. When the IPT was considered, both markers showed a significant increase in expression in the B compared to the T subtype (Figure 1B). The joint analysis of the IPT and HG showed significant variations in the expression of both markers for B-cell lymphoma according to the grade, which were not found in T-cell lymphomas (Figure 4 and Figure 5).

Immunohistochemical labeling with an antibody against iNOS was employed for the detection of M1 macrophages (Figure 1). In this case, the highest number of positively immunolabeled cells was found in low-grade (Figure 1A and Figure 2) and in B-cell lymphomas (Figure 1B and Figure 3). The joint analysis for the IPT and HG showed a significant increase in the number of iNOS positively immunostained cells among high-grade T-cell lymphomas and low-grade B-cell lymphomas (Figure 4 and Figure 5).

The Spearman test showed a positive correlation between the number of Iba1 and MAC387, CD204 and CD163 immunolabeled cells. The same positive correlation was observed between the number of cells immunostained with CD204 and CD163 markers, whereas a negative correlation was detected only between cells immunolabeled for CD204 and iNOS (Table 3). No significant correlation was observed between the rest of the markers.

## 4. Discussion

This study has evaluated the presence and distribution of macrophages in histological samples of canine lymphomas, where it has been demonstrated that they are present, in different amounts, with variations according to the HG and IPT. In relation to the samples analyzed, it has to be considered that B-cell lymphomas are reported as the most frequent in dogs, representing approximately 60–70% of the cases, compared to T-cell lymphomas, which constitute 30–40% [42]. However, in this study, from the 25 cases analyzed, the majority were T-cell lymphomas (14 vs. 11 of B-cell). This disagreement could be possibly due to the size of the sample examined. Regarding the HG, the majority of canine lymphomas show a high grade [42], while in our study, they were predominantly of low grade (7 vs. 18). This difference may be due, as already mentioned, to the number of samples analyzed, or also to the degree of progress of the tumor, since this finding may vary depending on the sample and the evolution of the neoplasia [42].

According to our results, macrophages seem to be an important cell type in canine lymphomas since its presence has been demonstrated in all the cases examined, where they constitute a heterogeneous population that showed variations related to the IPT and HG. To the best of our knowledge, this issue has not been investigated so far in canine lymphomas in spite of the increasing interest of the function that these cells could play in the pathogenesis and prognosis of haematopoietic tumors [26]. Macrophages have been shown to play an important role in the development of many tumors by influencing the microenvironment in which neoplastic cells are placed [4,24]. Although macrophages initially perform an antitumoral function (M1 macrophages) and phagocyte the apoptotic cells of the tumor, this process can induce the activation of a pro-oncogenic transcriptional profile, changing the phenotype of macrophages to an M2 type, which carry out an anti-inflammatory action, favoring tumor growth through the release of growth factors and inhibiting the antitumoral immune response [11,43].

Regarding the immunohistochemical characterization of the TAM, high-grade lymphomas showed the highest number of Iba1 positively immunolabeled cells, together with higher amounts of recently recruited (MAC387+) and M2 macrophages (CD204+ and CD163+). This was an expected result since high-grade lymphomas show a more aggressive biological behavior and a worse prognosis [30], and the presence of a greater infiltrate of TAM with a predominantly M2 phenotype has been correlated with a poor outcome in human lymphomas [14,17,44]. M2 macrophages seem to favor the invasion and progression of human lymphomas by remodeling the extracellular matrix [13], a mechanism that should be studied in canine species. One of the pathways of polarization of macrophages to the M2 phenotype is phagocytosis of the apoptotic bodies resulting from apoptosis, a phenomenon that increases together with the mitotic activity of the tumor [11,43]. Our results support this hypothesis since the highest number of M2 macrophages has been found in high-grade lymphomas where large amounts of tingible body macrophages linked to apoptosis are present. In contrast, low-grade lymphomas showed a higher number of M1 macrophages (iNOS+) that are related to an antitumoral activity [23]. The association between a high HG and the presence of large numbers of MAC387 positively immunostained macrophages suggests that this group of lymphomas is continuously recruiting monocytes from the circulation at the same time that high rates of apoptosis occur and, therefore, explaining why a higher number of macrophages are present [9,10].

In some studies, it was reported that CD163 is not a specific marker for M2 macrophages but, in the current work, its expression in terms of positively immunostained cells was similar to CD204, which has been considered as more specific for this macrophage subtype [45].

Regarding the immunophenotype, the number of TAM was higher in type B lymphomas, suggesting that these cells could play a role in the biology of this neoplasm in contrast to T-cell lymphomas. Moreover, the highest number of M1 macrophages was also found in B-cell lymphomas and might be related to the less aggressive behavior that this type of tumor shows with respect to T-cell lymphomas, which generally have a worse prognosis [16] and could be due to the already demonstrated antitumoral role of M1 macrophages [11,43]. However, it has to be considered that there was also a high count of M2 macrophages associated with a protumoral activity [11] and, therefore, not supporting the previous hypothesis. Probably the balance between the number and activity of both populations, occurring in each particular case of lymphoma, would determine its final functional outcome. Moreover, the role of other components of the tumor microenvironment such as fibroblasts, endothelial cells or cytokines produced by neoplastic or other cells could also play a role [46,47,48]. In line with this, T lymphocytes produce tumor necrosis factor alpha (TNF-α), which can induce the apoptosis of macrophages, decreasing their accumulation in the tumor [49] and explaining the high level of macrophage turnover that has been confirmed in T-cell lymphomas in contrast to B-cell lymphomas, although the total number of TAM was higher in the latter.

When analyzing the HG together with the IPT, it was observed that high-grade T-cell lymphomas showed a greater number of M1 macrophages than the low-grade ones, so it is likely to hypothesize that in this type of lymphomas there would be other factors, in addition to macrophages, influencing their more aggressive biological behavior, despite the antitumoral function of M1. The presence of a more inflammatory microenvironment in high-grade T-cell lymphomas in contrast with the low-grade ones, usually with an indolent clinical course [30], could be a possible explanation. In contrast, B-cell lymphomas showed the same pattern of macrophage distribution according to the grade, as already explained, regardless of the IPT. In this sense, in Hodgkin-type lymphomas there is a marked associated inflammation due to the release of proinflammatory cytokines produced by Reed–Sternberg cells and similarly it could occur in this case [50]. On the other hand, the fact that T lymphocytes can induce a Th1 response (by the production of interferon gamma), which could increase the polarization of TAM to a proinflammatory function (M1 subtype) [51] should be also considered, as well as the possibility that the presence of more amounts of M1 macrophages could antagonize the M2 function, since the production of TNF-α, a proinflammatory cytokine, could induce M2 macrophage apoptosis and a decrease in their numbers [49,51].

In human medicine, the presence of a high number of macrophages in lymphomas has been associated with a worse prognosis [7,17,52,53]. This fact could have not been corroborated in this study because the prognosis was not assessed. In any case, as far as we are aware, no other previous works have dealt with the role of macrophages in canine lymphomas and, therefore, further studies focusing on their influence over the clinical aspects such as prognosis, target therapies, etc., should be performed.

Finally, it is important to note that this study’s main limitations were the size of the study population and its retrospective nature. However, the results obtained are backed by the analysis carried out that was able to evaluate the main factors assessed that can influence the numbers of macrophages immunostained by the antibodies employed in canine nodal lymphomas and, therefore, can contribute to the understanding of the role of this particular type of cell in this type of tumor, where their presence or subtype characterization has been scarcely evaluated.

## 5. Conclusions

In conclusion, this study has demonstrated that a significant population of macrophages is present in canine lymphomas with variations in the amount and cell subtypes in relation to the HG and IPT. In those with a high grade, macrophages are actively recruited and show a predominant M2 phenotype associated with a protumoral activity, in contrast to low-grade lymphomas with lower numbers of macrophages and a predominant M1 subtype. When the IPT was analyzed, B-cell lymphomas had a higher number of macrophages, but T-cell lymphomas showed a greater recruitment, so it is possible that both macrophage subtypes could play a variable role according to the HG and IPT. The current work represents a first step in the assessment of the role of macrophages in canine lymphomas. Further studies including large numbers of cases, the use of other methodologies, such as additional immunohistochemical markers or flow cytometry techniques, or the evaluation of cytokines and chemokines that may influence the biological behavior of canine lymphomas would be necessary in order to confirm these results, considering also the complexity of the macrophage populations, including those present in neoplastic tissues.

## Figures and Tables

**Table 1:** Primary antibodies used in the study.

Primary Ab	Origin	Targeted Cell	Dilution	Epitope Retrieval
CD20 ThermoFisher Scientific (Waltham, MA, USA)	Rabbit (polyclonal)	B-cells	1:200	No
CD3 Agilent (Glostrup, Denmark)	Rabbit (polyclonal)	T-cells	1:300	Heat-induced pH 6, 90 min
Iba1 Wako (Richmond, VA, USA)	Rabbit (polyclonal)	Macrophages	1:2000	Heat-induced pH 6, 90 min
MAC387 Agilent (Glostrup, Denmark)	Mouse (monoclonal)	Monocytes and macrophages	1:200	Heat-induced pH 9, 90 min
CD204 TransGenic (Kobe, Japan)	Mouse (monoclonal)	M2 macrophages	1:400	Heat-induced pH 6, 90 min
CD163 TransGenic (Kobe, Japan)	Mouse (monoclonal)	M2 macrophages	1:300	Heat-induced pH 6, 90 min
iNOSS anta Cruz Biotechnology (Santa Cruz, CA, USA)	Rabbit (polyclonal)	M1 macrophages	1:100	Heat-induced pH 6, 90 min

Ab, antibody. CD, cluster of differentiation.

**Table 2:** Results of the post-hoc Tukey’s Honestly Significant Difference test for all pairwise comparisons in the model classified with ΔAIC_C_ <2 for the number of macrophages observed with each marker.

Marker	Linear Hypotheses	Estimate	SE	Z Value	*p* Value
Iba1	IPT (T)—IPT (B) = 0	−0.370	0.049	−7.433	<0.05
	HG (L)—HG (H) = 0	−0.399	0.039	−10.082	<0.001
	IPT (T) *HG (H)—IPT (T) *HG (L) = 0	−0.330	0.044	−7.449	<0.001
	IPT (B) *HG (H)—IPT (B) *HG (L) = 0	0.399	0.040	10.082	<0.001
MAC387	IPT (T)—IPT (B) = 0	0.877	0.619	14.160	<0.001
	HG (L)—HG (H) = 0	−0.782	0.045	−17.270	<0.05
	IPT (T) *HG (H)—IPT (T) *HG (L) = 0	−0.041	0.061	−0.669	0.504
	IPT (B) *HG (H)—IPT (B) *HG (L) = 0	0.783	0.045	17.272	<0.05
CD204	IPT (T)—IPT (B) = 0	−0.329	0.047	−7.046	<0.05
	HG (L)—HG (H) = 0	−0.435	0.038	−11.440	<0.001
	IPT (T) *HG (H)—IPT (T) *HG (L) = 0	−0.011	0.043	−0.260	0.994
	IPT (B) *HG (H)—IPT (B) *HG (L) = 0	−0.434	0.038	11.439	<0.001
CD163	IPT (T)—IPT (B) = 0	−0.190	0.057	−3.303	0.001
	HG (L)—HG (H) = 0	−0.557	0.050	−11.079	<0.001
	IPT (T) *HG (H)—IPT (T) *HG (L) = 0	0.052	0.051	1.022	0.307
	IPT (B) *HG (H)—IPT (B) *HG (L) = 0	0.557	0.050	11.079	<0.001
iNOS	IPT (T)—IPT (B) = 0	−0.892	0.099	−9.046	<0.05
	HG (L)—HG (H) = 0	0.383	0.093	4.094	<0.05
	IPT (T) *HG (H)—IPT (T) *HG (L) = 0	0.540	0.071	7.599	<0.01
	IPT (B) *HG (H)—IPT (B) *HG (L) = 0	−0.383	0.093	−4.094	<0.05

SE, standard error. IPT, immunophenotype. HG, histological grade. *, interaction.

**Table 3:** Spearman correlation test.

Marker	Iba1	MAC387	CD204	CD163	iNOS
Iba1		0.397 *	0.725 ***	0.668 ***	−0.205
MAC387			0.252	0.362	0.118
CD204				0.572 **	−0.449 *
CD163					−0.250

*, *p* < 0.05. **, *p* < 0.01. ***, *p* < 0.001.

## Data Availability

The data supporting the conclusions of this article will be made available by the authors, under reasonable request.

## Supplementary Materials

The following are available online at https://www.mdpi.com/article/10.3390/ani11082301/s1, Table S1: model selection for the effect of immunophenotype and the histological grade in the number of different macrophage subtypes detected in canine lymph node lymphomas.
