# Supplementary material for: Immunohistochemical Characterization of Tumor-Associated Macrophages in Canine Lymphomas
Source: Animals (Basel). 2021 Aug 4;11(8):2301. doi: 10.3390/ani11082301 (PMC8388421; doi:10.3390/ani11082301)
Supplement: Supplementary file 1 [file animals-11-02301-s001.zip › animals-1292789-supplementary.pdf]

Supplementary Material

Table S1: Model selection for the effect of IPT and the HG in the number of different macrophage subtypes detected in canine lymph node lymphomas.

Marker	Biological model	K	AIC_c	ΔAIC_c	W_i	R^2 (adj)
**Iba1**	HG+IPT+HG*IPT	4	1153.4	0.00	1.00	0.657
	HG+IPT	3	1307.2	153.83	0.00	0.593
	IPT	2	1307.5	154.14	0.00	0.272
	Null model	1	1331.9	178.53	0.00	<0.001
	HG	2	1333.6	180.21	0.00	0.162
**MAC387**	HG+IPT+HG*IPT	4	1846.6	0.00	1.00	0.997
	HG+IPT	3	1969.3	122.69	0.00	0.994
	HG	2	2071.2	224.61	0.00	0.980
	IPT	2	2141.0	294.41	0.00	0.873
	Null model	1	2298.8	452.14	0.00	<0.001
**CD204**	HG+IPT+HG*IPT	4	1588.0	0.00	1.00	0.996
	HG	2	1646.5	58.48	0.00	0.943
	HG+IPT	3	1647.6	59.64	0.00	0.946
	IPT	2	1711.3	123.30	0.00	0.237
	Null model	1	1715.7	127.69	0.00	0.000
**CD163**	HG+IPT+HG*IPT	4	1430.6	0.00	1.00	0.994
	HG+IPT	3	1478.2	47.61	0.00	0.957
	HG	2	1491.7	61.09	0.00	0.918
	IPT	2	1547.4	116.74	0.00	0.240
	Null model	1	1551.9	121.25	0.00	<0.001
**iNOS**	HG+IPT+HG*IPT	4	668.1	0.00	1.00	0.973
	HG+IPT	3	728.6	60.47	0.00	0.668
	IPT	2	733.9	65.83	0.00	0.545
	HG	2	749.4	81.32	0.00	0.153
	Null model	1	751.2	83.12	0.00	<0.001

IPT, immunophenotype. HG, histological grade. *, interaction. +, additive effect. K, number of estimated parameters. AIC_c, Akaike's Information Criterion corrected for small sample size. ΔAIC_c, difference of AICc between the model and the most-parsimonious model. W_i, Akaike weight of the model. R^2 (adj), adjusted R2 of the fitted model (see Burnham and Anderson, 2002 and Zuur, 2007).
